# Emotional Intelligence and Psychological Distress as determinants of Quality of Life in Healthcare Professionals

**DOI:** 10.1192/j.eurpsy.2026.10623

**Published:** 2026-07-31

**Authors:** M. Theodoratou, A. Margaroni, M. Sofologi, A. Argyriadi, E. Efthymiou, A. Argyriadis

**Affiliations:** 1 Social Sciences, Hellenic Open University, Patras, Greece; 2 Social Sciences and Art and Humanistic Studies, Neapolis University Pafos, Pafos, Cyprus; 3 University of Ioannina, Ioannina, Greece; 4 Frederick University, Nicosia, Cyprus; 5 University of Zayed, Abou Dhabi, United Arab Emirates; 6 Nursing, Hellenic Mediterannean University, Herakleion, Greece

## Abstract

**Introduction:**

Healthcare professionals are frequently exposed to high levels of stress, emotional strain, and demanding work conditions, which may lead to psychological distress and burnout. Emotional intelligence (EI), defined as the ability to recognize, regulate, and use emotions effectively, is considered a protective factor that enhances resilience, reduces stress, and improves quality of life (QoL). Understanding the interplay between EI, psychological distress, and QoL is crucial for safeguarding the well-being and professional effectiveness of healthcare workers.

**Objectives:**

This study aimed to investigate the relationships between emotional intelligence, psychological distress, and quality of life among healthcare professionals. Specifically, it examined: (1) the effect of EI on stress management and resilience; (2) the role of EI as a protective factor against psychological distress; and (3) the extent to which EI contributes to improved quality of life.

**Methods:**

A cross-sectional study was conducted with a random sample of 250 healthcare professionals from hospital settings (82.8% women, mean age = 41.9 years, SD = 10.5). Participants represented various specialties, including physicians, nurses, therapists, and administrative staff. Data were collected using standardized questionnaires: the NHS Emotional Intelligence Questionnaire (five EI subscales), the Kessler K10 Scale (psychological distress), and the ProQOL Scale (quality of professional life).

**Results:**

The mean EI subscale scores indicated relatively high levels of self-awareness (M = 38.9) and empathy (M = 36.7), but lower emotion regulation (M = 33.2). Psychological distress scores averaged 30.2, reflecting moderate risk levels. Professional quality of life showed high compassion satisfaction (M = 37.8), low burnout (M = 24.4), and moderate secondary traumatic stress (M = 30.6). Strong positive correlations were found among EI dimensions (r = 0.79–0.84, p < 0.01), while EI was negatively correlated with psychological distress (r = -0.52, p < 0.01) and burnout (r = -0.49, p < 0.01). EI was positively associated with overall QoL (r = 0.58, p < 0.01). Marital status, however, was associated with higher self-awareness and lower distress (p < 0.05).

**Conclusions:**

This study highlights the critical role of emotional intelligence in mitigating psychological distress and enhancing quality of life among healthcare professionals. Higher EI, particularly in self-awareness, empathy, and self-motivation, was associated with reduced stress, lower burnout, and greater professional satisfaction. Interventions that cultivate EI—through training, supervision, and supportive workplace cultures—may strengthen resilience, reduce the risk of compassion fatigue, and improve the overall well-being of healthcare staff. Future research should explore longitudinal effects and intervention strategies to sustain mental health in healthcare settings.

**Disclosure of Interest:**

None Declared

